# *N*-{4-[3-(4-Fluoro­phen­yl)pyrido[2,3-*b*]pyrazin-2-yl]-2-pyrid­yl}isopropyl­amine

**DOI:** 10.1107/S1600536809038173

**Published:** 2009-09-26

**Authors:** Pierre Koch, Dieter Schollmeyer, Stefan Laufer

**Affiliations:** a Institute of Pharmacy, Department of Pharmaceutical and Medicinal Chemistry, Eberhard-Karls-University Tübingen, Auf der Morgenstelle 8, 72076 Tübingen, Germany; b Department of Organic Chemistry, Johannes Gutenberg-University Mainz, Duesbergweg 10-14, D-55099 Mainz, Germany

## Abstract

In the crystal structure of the title compound, C_21_H_18_FN_5_, the pyridopyrazine ring system forms dihedral angles of 33.27 (7) and 48.69 (9)° with the 4-fluoro­phenyl and pyridine ring, respectively. The dihedral angle of the 4-fluoro­phenyl and pyridine rings is 57.45 (8)°. The crystal packing is characterized by an inter­molecular N—H⋯N hydrogen bond.

## Related literature

For the preparation of pyridopyrazines under microwave conditions, see: Zhao *et al*. (2004 ▶)
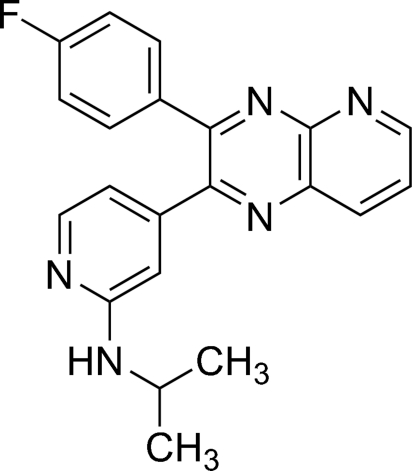


## Experimental

### 

#### Crystal data

C_21_H_18_FN_5_
*M* *_r_* = 359.40
Monoclinic, 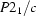

*a* = 12.042 (1) Å
*b* = 7.6586 (2) Å
*c* = 20.095 (2) Å
β = 100.215 (5)°
*V* = 1823.8 (3) Å^3^
*Z* = 4
Cu *K*α radiation
μ = 0.72 mm^−1^
*T* = 193 K
0.50 × 0.20 × 0.10 mm

#### Data collection

Enraf–Nonius CAD-4 diffractometer
Absorption correction: ψ scan (*CORINC*; Dräger & Gattow, 1971 ▶) *T*_min_ = 0.743, *T*_max_ = 0.998
3578 measured reflections
3468 independent reflections
2912 reflections with *I* > 2σ(*I*)
*R*_int_ = 0.063
3 standard reflections frequency: 60 min intensity decay: 2%

#### Refinement

*R*[*F* ^2^ > 2σ(*F* ^2^)] = 0.047
*wR*(*F* ^2^) = 0.127
*S* = 1.05
3468 reflections
247 parameters
H-atom parameters constrained
Δρ_max_ = 0.29 e Å^−3^
Δρ_min_ = −0.28 e Å^−3^

### 

Data collection: *CAD-4 Software* (Enraf–Nonius, 1989 ▶); cell refinement: *CAD-4 Software*; data reduction: *CORINC* (Dräger & Gattow, 1971 ▶); program(s) used to solve structure: *SIR97* (Altomare *et al.*, 1999 ▶); program(s) used to refine structure: *SHELXL97* (Sheldrick, 2008 ▶); molecular graphics: *PLATON* (Spek, 2009 ▶); software used to prepare material for publication: *PLATON*.

## Supplementary Material

Crystal structure: contains datablocks I, global. DOI: 10.1107/S1600536809038173/im2142sup1.cif

Structure factors: contains datablocks I. DOI: 10.1107/S1600536809038173/im2142Isup2.hkl

Additional supplementary materials:  crystallographic information; 3D view; checkCIF report

## Figures and Tables

**Table 1:** Hydrogen-bond geometry (Å, °)

*D*—H⋯*A*	*D*—H	H⋯*A*	*D*⋯*A*	*D*—H⋯*A*
N17—H17⋯N6^i^	0.91	2.32	3.166 (2)	154

Symmetry code: (i) 
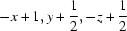
.

**Fig. 1.**
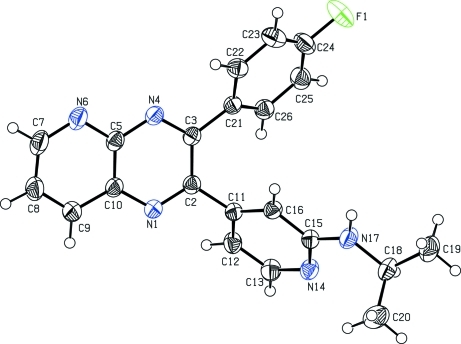
View of compound I. Displacement ellipsoids are drawn at the 50% probability level.

## supplementary crystallographic information

### Comment

The title compound, {4-[3-(4-fluorophenyl)-pyrido[2,3-*b*]pyrazin-2-yl]-pyridin-2-yl}-isopropylamine (I), was prepared in the course of our studies on pyridin-4-yl-substituted pyridopyrazines as p38 mitogen-activated protein (MAP) kinase inhibitors.

The microwave-assisted reaction of 1-(4-fluorophenyl)-2-(2-(isopropylamino)-pyridin-4-yl)ethane-1,2-dione and 2,3-diaminopyridine yields two regioisomers, {4-[3-(4-fluorophenyl)-pyrido[2,3-*b*]pyrazin-2-yl]-pyridin-2-yl}-isopropylamine (I) and {4-[2-(4-fluorophenyl)-pyrido[3,2-*b*]pyrazin-3-yl]-pyridin-2-yl}-isopropylamine (II). The isomers were separated by flash-chromatography. To identify the two regioisomers *X*-ray crystallography was used. Herein we present the *X*-ray crystallographic data of the first eluted isomer I.

As might be expected the 4-fluorophenyl, the pyridine ring as well as the pyridopyrazine ring are planar (Figure 1). The pyridopyrazine ring encloses dihedral angles of 33.27 (7)° and 48.69 (9)° to the 4-fluorophenyl ring and the pyridine ring, respectively. The dihedral angle of the 4-fluorophenyl ring and the pyridine ring measures to 57.45 (8)° to the pyridine ring. The crystal packing is characterized by an intermolecular hydrogen bond N17–H17···N6 2.32 Å.

### Experimental

1-(4-Fluorophenyl)-2-(2-(isopropylamino)pyridin-4-yl)ethane-1,2-dione (115 mg, 0.4 mmol), 2,3-diaminopyridine (44 mg, 0.4 mmol), and methanol/glacial acetic acid (3 ml, 9:1, *v:v*) were combined in a reaction vial. The reaction vessel was heated in a microwave reactor for 5 min at 433 K (initial power 250 W). Then a stream of compressed air cooled the reaction vessel to r.t. The solvent was removed under reduced pressure and the residue was purified by flash-chromatography (silica gel, light petroleum /ethyl acetate 1:1 to 1:4) to yield 46 mg (33%) of I as a yellow solid. Suitable crystals of compound I for X-ray diffraction were obtained from a solution in *n*-hexane - diethyl ether (2:1) by slow evaporation of the solvent at 298 K.

### Refinement

Hydrogen atoms attached to carbons were placed at calculated positions with C—H = 0.95 Å (aromatic) or 0.98–0.99 Å (*sp*^3^ C-atom). Hydrogen atom attached to N17 was located in diff. Fourier maps. All H atoms were refined in the riding-model approximation with isotropic displacement parameters (set at 1.2–1.5 times of the *U*_eq_ of the parent atom).

### Crystal data

**Table:**

C_21_H_18_FN_5_	*F*(000) = 752
*M**_r_* = 359.40	*D*_x_ = 1.309 Mg m^−^^3^
Monoclinic, *P*2_1_/*c*	Cu *K*α radiation, λ = 1.54178 Å
Hall symbol: -P 2ybc	Cell parameters from 25 reflections
*a* = 12.042 (1) Å	θ = 60–69°
*b* = 7.6586 (2) Å	µ = 0.72 mm^−^^1^
*c* = 20.095 (2) Å	*T* = 193 K
β = 100.215 (5)°	Plate, yellow
*V* = 1823.8 (3) Å^3^	0.50 × 0.20 × 0.10 mm
*Z* = 4	

### Data collection

**Table:**

Enraf–Nonius CAD-4 diffractometer	2912 reflections with *I* > 2σ(*I*)
Radiation source: rotating anode	*R*_int_ = 0.063
graphite	θ_max_ = 70.2°, θ_min_ = 3.7°
ω/2θ scans	*h* = −14→14
Absorption correction: ψ scan (CORINC; Dräger & Gattow, 1971)	*k* = −9→0
*T*_min_ = 0.743, *T*_max_ = 0.998	*l* = 0→24
3578 measured reflections	3 standard reflections every 60 min
3468 independent reflections	intensity decay: 2%

### Refinement

**Table:**

Refinement on *F*^2^	Secondary atom site location: difference Fourier map
Least-squares matrix: full	Hydrogen site location: inferred from neighbouring sites
*R*[*F*^2^ > 2σ(*F*^2^)] = 0.047	H-atom parameters constrained
*wR*(*F*^2^) = 0.127	*w* = 1/[σ^2^(*F*_o_^2^) + (0.065*P*)^2^ + 0.5513*P*] where *P* = (*F*_o_^2^ + 2*F*_c_^2^)/3
*S* = 1.05	(Δ/σ)_max_ = 0.001
3468 reflections	Δρ_max_ = 0.29 e Å^−^^3^
247 parameters	Δρ_min_ = −0.28 e Å^−^^3^
0 restraints	Extinction correction: *SHELXL97* (Sheldrick, 2008), Fc^*^=kFc[1+0.001xFc^2^λ^3^/sin(2θ)]^-1/4^
Primary atom site location: structure-invariant direct methods	Extinction coefficient: 0.0038 (4)

### Special details

**Table:**

Geometry. All e.s.d.'s (except the e.s.d. in the dihedral angle between two l.s. planes) are estimated using the full covariance matrix. The cell e.s.d.'s are taken into account individually in the estimation of e.s.d.'s in distances, angles and torsion angles; correlations between e.s.d.'s in cell parameters are only used when they are defined by crystal symmetry. An approximate (isotropic) treatment of cell e.s.d.'s is used for estimating e.s.d.'s involving l.s. planes.
Refinement. Refinement of *F*^2^ against ALL reflections. The weighted *R*-factor *wR* and goodness of fit *S* are based on *F*^2^, conventional *R*-factors *R* are based on *F*, with *F* set to zero for negative *F*^2^. The threshold expression of *F*^2^ > σ(*F*^2^) is used only for calculating *R*-factors(gt) *etc*. and is not relevant to the choice of reflections for refinement. *R*-factors based on *F*^2^ are statistically about twice as large as those based on *F*, and *R*- factors based on ALL data will be even larger.

### Fractional atomic coordinates and isotropic or equivalent isotropic displacement parameters (Å^2^)

**Table:**

	*x*	*y*	*z*	*U*_iso_*/*U*_eq_	
F1	0.34953 (12)	0.36820 (19)	0.45738 (6)	0.0682 (4)	
N1	0.47943 (11)	0.38937 (19)	0.08886 (7)	0.0343 (3)	
C2	0.43429 (13)	0.4033 (2)	0.14386 (8)	0.0292 (3)	
C3	0.50235 (12)	0.38498 (19)	0.21015 (8)	0.0278 (3)	
N4	0.61263 (11)	0.36734 (17)	0.21777 (7)	0.0315 (3)	
C5	0.66035 (13)	0.3611 (2)	0.16141 (8)	0.0319 (4)	
N6	0.77471 (11)	0.3488 (2)	0.17218 (8)	0.0412 (4)	
C7	0.82147 (15)	0.3384 (3)	0.11753 (10)	0.0463 (5)	
H7	0.9014	0.3315	0.1237	0.056*	
C8	0.76109 (16)	0.3370 (3)	0.05108 (10)	0.0508 (5)	
H8	0.7998	0.3258	0.0140	0.061*	
C9	0.64671 (16)	0.3516 (3)	0.04001 (9)	0.0470 (5)	
H9	0.6042	0.3522	−0.0046	0.056*	
C10	0.59296 (14)	0.3660 (2)	0.09677 (8)	0.0336 (4)	
C11	0.31151 (12)	0.4444 (2)	0.13098 (7)	0.0287 (3)	
C12	0.23734 (13)	0.3532 (2)	0.08126 (8)	0.0349 (4)	
H12	0.2628	0.2571	0.0581	0.042*	
C13	0.12700 (13)	0.4069 (2)	0.06713 (8)	0.0370 (4)	
H13	0.0772	0.3442	0.0336	0.044*	
N14	0.08393 (10)	0.54181 (18)	0.09720 (7)	0.0345 (3)	
C15	0.15497 (13)	0.6273 (2)	0.14529 (8)	0.0307 (3)	
C16	0.26989 (12)	0.5820 (2)	0.16339 (8)	0.0299 (3)	
H16	0.3181	0.6456	0.1975	0.036*	
N17	0.11431 (11)	0.76489 (19)	0.17634 (7)	0.0382 (4)	
H17	0.1587	0.8153	0.2129	0.046*	
C18	−0.00040 (14)	0.8299 (2)	0.15770 (9)	0.0393 (4)	
H18	−0.0531	0.7279	0.1513	0.047*	
C19	−0.02796 (18)	0.9414 (3)	0.21535 (11)	0.0545 (5)	
H19A	−0.0258	0.8687	0.2557	0.082*	
H19B	−0.1035	0.9917	0.2022	0.082*	
H19C	0.0276	1.0356	0.2252	0.082*	
C20	−0.0150 (2)	0.9323 (3)	0.09207 (11)	0.0610 (6)	
H20A	0.0307	1.0388	0.0989	0.091*	
H20B	−0.0947	0.9636	0.0780	0.091*	
H20C	0.0094	0.8607	0.0569	0.091*	
C21	0.45692 (13)	0.3816 (2)	0.27448 (8)	0.0295 (3)	
C22	0.52397 (14)	0.4499 (2)	0.33258 (8)	0.0355 (4)	
H22	0.5953	0.4994	0.3296	0.043*	
C23	0.48813 (17)	0.4467 (2)	0.39438 (9)	0.0439 (4)	
H23	0.5330	0.4955	0.4337	0.053*	
C24	0.38569 (17)	0.3708 (3)	0.39702 (9)	0.0441 (4)	
C25	0.31854 (15)	0.2973 (2)	0.34194 (9)	0.0410 (4)	
H25	0.2491	0.2433	0.3460	0.049*	
C26	0.35451 (14)	0.3039 (2)	0.28003 (8)	0.0342 (4)	
H26	0.3088	0.2549	0.2411	0.041*	

### Atomic displacement parameters (Å^2^)

**Table:**

	*U*^11^	*U*^22^	*U*^33^	*U*^12^	*U*^13^	*U*^23^
F1	0.0892 (9)	0.0835 (9)	0.0395 (6)	−0.0090 (7)	0.0321 (6)	0.0019 (6)
N1	0.0320 (7)	0.0395 (8)	0.0321 (7)	0.0079 (6)	0.0075 (5)	0.0045 (6)
C2	0.0298 (8)	0.0263 (7)	0.0321 (8)	0.0022 (6)	0.0070 (6)	0.0014 (6)
C3	0.0282 (7)	0.0230 (7)	0.0322 (8)	0.0009 (6)	0.0054 (6)	0.0008 (6)
N4	0.0285 (7)	0.0311 (7)	0.0348 (7)	0.0033 (5)	0.0053 (5)	0.0024 (5)
C5	0.0292 (8)	0.0277 (8)	0.0399 (9)	0.0037 (6)	0.0091 (6)	0.0051 (6)
N6	0.0275 (7)	0.0448 (9)	0.0524 (9)	0.0048 (6)	0.0103 (6)	0.0048 (7)
C7	0.0305 (8)	0.0486 (11)	0.0634 (12)	0.0084 (8)	0.0181 (8)	0.0125 (9)
C8	0.0460 (10)	0.0590 (12)	0.0540 (11)	0.0162 (9)	0.0273 (9)	0.0193 (9)
C9	0.0438 (10)	0.0610 (12)	0.0395 (9)	0.0169 (9)	0.0164 (8)	0.0154 (9)
C10	0.0324 (8)	0.0325 (8)	0.0373 (8)	0.0085 (6)	0.0101 (6)	0.0074 (7)
C11	0.0279 (7)	0.0302 (8)	0.0285 (7)	0.0022 (6)	0.0066 (6)	0.0025 (6)
C12	0.0333 (8)	0.0360 (9)	0.0363 (8)	0.0011 (7)	0.0085 (6)	−0.0077 (7)
C13	0.0306 (8)	0.0409 (9)	0.0386 (9)	−0.0044 (7)	0.0037 (7)	−0.0099 (7)
N14	0.0259 (6)	0.0389 (8)	0.0383 (7)	0.0003 (5)	0.0045 (5)	−0.0038 (6)
C15	0.0288 (7)	0.0327 (8)	0.0311 (7)	0.0017 (6)	0.0073 (6)	0.0008 (6)
C16	0.0276 (7)	0.0307 (8)	0.0305 (7)	0.0006 (6)	0.0026 (6)	−0.0011 (6)
N17	0.0308 (7)	0.0410 (8)	0.0409 (8)	0.0088 (6)	0.0018 (6)	−0.0093 (6)
C18	0.0299 (8)	0.0371 (9)	0.0503 (10)	0.0077 (7)	0.0057 (7)	−0.0037 (8)
C19	0.0490 (11)	0.0553 (12)	0.0597 (12)	0.0199 (9)	0.0104 (9)	−0.0105 (10)
C20	0.0668 (14)	0.0562 (13)	0.0567 (13)	0.0236 (11)	0.0025 (10)	0.0048 (10)
C21	0.0318 (8)	0.0264 (8)	0.0306 (8)	0.0049 (6)	0.0068 (6)	0.0033 (6)
C22	0.0389 (9)	0.0327 (8)	0.0344 (8)	−0.0004 (7)	0.0051 (7)	0.0014 (7)
C23	0.0570 (11)	0.0424 (10)	0.0310 (8)	−0.0020 (8)	0.0044 (8)	−0.0006 (7)
C24	0.0588 (11)	0.0448 (10)	0.0323 (9)	0.0049 (9)	0.0183 (8)	0.0058 (7)
C25	0.0415 (9)	0.0400 (9)	0.0446 (10)	0.0005 (8)	0.0163 (7)	0.0077 (8)
C26	0.0351 (8)	0.0320 (8)	0.0360 (8)	0.0019 (7)	0.0076 (6)	0.0015 (7)

### Geometric parameters (Å, °)

**Table:**

F1—C24	1.3595 (19)	C15—N17	1.359 (2)
N1—C2	1.3197 (19)	C15—C16	1.410 (2)
N1—C10	1.360 (2)	C16—H16	0.9500
C2—C3	1.442 (2)	N17—C18	1.454 (2)
C2—C11	1.489 (2)	N17—H17	0.9144
C3—N4	1.3165 (19)	C18—C20	1.518 (3)
C3—C21	1.491 (2)	C18—C19	1.523 (2)
N4—C5	1.359 (2)	C18—H18	1.0000
C5—N6	1.359 (2)	C19—H19A	0.9800
C5—C10	1.405 (2)	C19—H19B	0.9800
N6—C7	1.322 (2)	C19—H19C	0.9800
C7—C8	1.403 (3)	C20—H20A	0.9800
C7—H7	0.9500	C20—H20B	0.9800
C8—C9	1.360 (3)	C20—H20C	0.9800
C8—H8	0.9500	C21—C26	1.391 (2)
C9—C10	1.412 (2)	C21—C22	1.398 (2)
C9—H9	0.9500	C22—C23	1.385 (2)
C11—C16	1.379 (2)	C22—H22	0.9500
C11—C12	1.403 (2)	C23—C24	1.373 (3)
C12—C13	1.372 (2)	C23—H23	0.9500
C12—H12	0.9500	C24—C25	1.370 (3)
C13—N14	1.346 (2)	C25—C26	1.389 (2)
C13—H13	0.9500	C25—H25	0.9500
N14—C15	1.342 (2)	C26—H26	0.9500
			
C2—N1—C10	117.88 (13)	C15—N17—C18	123.40 (14)
N1—C2—C3	120.98 (14)	C15—N17—H17	119.3
N1—C2—C11	114.55 (13)	C18—N17—H17	117.2
C3—C2—C11	124.44 (13)	N17—C18—C20	111.07 (15)
N4—C3—C2	120.92 (14)	N17—C18—C19	108.76 (15)
N4—C3—C21	114.53 (13)	C20—C18—C19	111.33 (16)
C2—C3—C21	124.54 (13)	N17—C18—H18	108.5
C3—N4—C5	118.27 (13)	C20—C18—H18	108.5
N4—C5—N6	115.86 (14)	C19—C18—H18	108.5
N4—C5—C10	120.63 (14)	C18—C19—H19A	109.5
N6—C5—C10	123.51 (15)	C18—C19—H19B	109.5
C7—N6—C5	116.14 (15)	H19A—C19—H19B	109.5
N6—C7—C8	124.47 (16)	C18—C19—H19C	109.5
N6—C7—H7	117.8	H19A—C19—H19C	109.5
C8—C7—H7	117.8	H19B—C19—H19C	109.5
C9—C8—C7	119.59 (17)	C18—C20—H20A	109.5
C9—C8—H8	120.2	C18—C20—H20B	109.5
C7—C8—H8	120.2	H20A—C20—H20B	109.5
C8—C9—C10	118.05 (17)	C18—C20—H20C	109.5
C8—C9—H9	121.0	H20A—C20—H20C	109.5
C10—C9—H9	121.0	H20B—C20—H20C	109.5
N1—C10—C5	121.04 (14)	C26—C21—C22	118.82 (15)
N1—C10—C9	120.74 (15)	C26—C21—C3	122.93 (14)
C5—C10—C9	118.19 (15)	C22—C21—C3	118.12 (14)
C16—C11—C12	118.58 (14)	C23—C22—C21	121.09 (16)
C16—C11—C2	120.82 (14)	C23—C22—H22	119.5
C12—C11—C2	120.37 (14)	C21—C22—H22	119.5
C13—C12—C11	117.96 (15)	C24—C23—C22	117.83 (16)
C13—C12—H12	121.0	C24—C23—H23	121.1
C11—C12—H12	121.0	C22—C23—H23	121.1
N14—C13—C12	124.99 (15)	F1—C24—C25	118.57 (17)
N14—C13—H13	117.5	F1—C24—C23	118.20 (17)
C12—C13—H13	117.5	C25—C24—C23	123.23 (16)
C15—N14—C13	116.76 (13)	C24—C25—C26	118.40 (16)
N14—C15—N17	118.24 (14)	C24—C25—H25	120.8
N14—C15—C16	122.48 (14)	C26—C25—H25	120.8
N17—C15—C16	119.27 (14)	C25—C26—C21	120.58 (16)
C11—C16—C15	119.22 (14)	C25—C26—H26	119.7
C11—C16—H16	120.4	C21—C26—H26	119.7
C15—C16—H16	120.4		
			
C10—N1—C2—C3	−3.3 (2)	C2—C11—C12—C13	−174.10 (15)
C10—N1—C2—C11	174.81 (14)	C11—C12—C13—N14	0.3 (3)
N1—C2—C3—N4	5.3 (2)	C12—C13—N14—C15	−1.1 (3)
C11—C2—C3—N4	−172.55 (14)	C13—N14—C15—N17	179.56 (15)
N1—C2—C3—C21	−173.81 (14)	C13—N14—C15—C16	1.1 (2)
C11—C2—C3—C21	8.3 (2)	C12—C11—C16—C15	−0.5 (2)
C2—C3—N4—C5	−2.1 (2)	C2—C11—C16—C15	174.11 (14)
C21—C3—N4—C5	177.08 (13)	N14—C15—C16—C11	−0.4 (2)
C3—N4—C5—N6	177.84 (14)	N17—C15—C16—C11	−178.81 (15)
C3—N4—C5—C10	−2.6 (2)	N14—C15—N17—C18	−3.3 (2)
N4—C5—N6—C7	178.44 (16)	C16—C15—N17—C18	175.18 (15)
C10—C5—N6—C7	−1.1 (2)	C15—N17—C18—C20	−75.1 (2)
C5—N6—C7—C8	−0.9 (3)	C15—N17—C18—C19	162.02 (17)
N6—C7—C8—C9	1.8 (3)	N4—C3—C21—C26	−143.17 (15)
C7—C8—C9—C10	−0.7 (3)	C2—C3—C21—C26	36.0 (2)
C2—N1—C10—C5	−1.5 (2)	N4—C3—C21—C22	32.6 (2)
C2—N1—C10—C9	−179.42 (16)	C2—C3—C21—C22	−148.19 (15)
N4—C5—C10—N1	4.7 (2)	C26—C21—C22—C23	−2.3 (2)
N6—C5—C10—N1	−175.85 (15)	C3—C21—C22—C23	−178.29 (15)
N4—C5—C10—C9	−177.38 (16)	C21—C22—C23—C24	1.4 (3)
N6—C5—C10—C9	2.1 (3)	C22—C23—C24—F1	−179.53 (16)
C8—C9—C10—N1	176.83 (18)	C22—C23—C24—C25	0.6 (3)
C8—C9—C10—C5	−1.1 (3)	F1—C24—C25—C26	178.49 (16)
N1—C2—C11—C16	−128.17 (16)	C23—C24—C25—C26	−1.7 (3)
C3—C2—C11—C16	49.8 (2)	C24—C25—C26—C21	0.7 (3)
N1—C2—C11—C12	46.3 (2)	C22—C21—C26—C25	1.2 (2)
C3—C2—C11—C12	−135.71 (16)	C3—C21—C26—C25	177.00 (15)
C16—C11—C12—C13	0.5 (2)		

### Hydrogen-bond geometry (Å, °)

**Table:**

*D*—H···*A*	*D*—H	H···*A*	*D*···*A*	*D*—H···*A*
N17—H17···N6^i^	0.91	2.32	3.166 (2)	154

Symmetry codes: (i) −*x*+1, *y*+1/2, −*z*+1/2.

## References

[bb1] Altomare, A., Burla, M. C., Camalli, M., Cascarano, G. L., Giacovazzo, C., Guagliardi, A., Moliterni, A. G. G., Polidori, G. & Spagna, R. (1999). *J. Appl. Cryst.* **32**, 115–119.

[bb2] Dräger, M. & Gattow, G. (1971). *Acta Chem. Scand.* **25**, 761–762.

[bb3] Enraf–Nonius (1989). *CAD-4 Software* Enraf–Nonius, Delft, The Netherlands.

[bb4] Sheldrick, G. M. (2008). *Acta Cryst.* A**64**, 112–122. 10.1107/S0108767307043930 18156677

[bb5] Spek, A. L. (2009). *Acta Cryst.* D**65**, 148–155. 10.1107/S090744490804362X PMC2631630 19171970

[bb6] Zhao, Z., Wisnoski, D. D., Wolkenberg, S. E., Leister, W. H., Wang, Y. & Lindsley, C. W. (2004). *Tetrahedron Lett.* **45**, 4873–4876.

